# Relationships between smoking behavior, systemic inflammation, and myocardial infarction and their effects on long‐term outcomes in older Chinese patients with coronary artery disease: A prospective study with a 10‐year follow‐up

**DOI:** 10.1002/mco2.240

**Published:** 2023-04-27

**Authors:** Nanhang Lei, Yijie Sun, Hanwang Zhou, Qiong Liu, Yali Zhao, Pengbin Yin, Ping Ping, Shihui Fu

**Affiliations:** ^1^ Hainan Hospital of Chinese People's Liberation Army General Hospital Sanya China; ^2^ Chinese People's Liberation Army General Hospital Beijing China; ^3^ General Station for Drug and Instrument Supervision and Control Beijing China

Dear Editor,

Coronary artery disease (CAD) is one of the most significant cardiovascular diseases that seriously affects human health worldwide and can cause a huge economic burden.^1^ Research on older people with CAD requires more attention because the aging population is increasing rapidly. The pathological process of CAD is thought to involve atherosclerosis, and inflammation plays a key role in CAD. C‐reactive protein (CRP) is a sensitive and stable marker of systemic inflammation and has been postulated to predict the extent of atherosclerosis.^2^ CRP contributes to all phases of atherosclerosis, exerts anti‐inflammatory effects, and maintains balance in the inflammatory process.^2^ CRP levels that are slightly increased for a prolonged period reflect a low level of persistent inflammation in the body, thus characterizing CRP as a predictor of adverse outcomes.^2^ However, reports are lacking on the relationship between elevated CRP levels and long‐term outcomes in older patients with CAD.

Smoking behavior is an important risk factor for CAD. Among smokers, two‐thirds of deaths due to CAD were attributable to a smoking age of 55−74 years old.^3^ Moreover, smoking has been suggested to aggravate systemic inflammation in the body.^3^ However, the relationships among smoking behavior, systemic inflammation, acute myocardial infarction (AMI), and long‐term outcomes in older patients with CAD need to be elucidated. This prospective study aimed to examine the relationships between smoking behavior, elevated CRP levels, and AMI in older Chinese patients with CAD and their effects on long‐term outcomes in these patients after a 10‐year follow‐up.

This study consecutively included 987 patients with CAD who were ≥60 years old and were admitted to the Department of Geriatric Cardiology, Chinese People's Liberation Army (PLA) General Hospital (Figure 1A). According to the guidelines of the American College of Cardiology, American Heart Association, and European College of Cardiology, the diagnosis of CAD was made by the chief physicians on the basis of clinical histories, angina symptoms, cardiac markers, and auxiliary examinations, including electrocardiogram (rest and exercise), echocardiography, radionuclide imaging, computed tomography, and coronary angiography. Chinese PLA General Hospital provided long‐term and comprehensive medical services and kept clinical information and death records, thus making it easier for us to follow these patients effectively and judge the endpoints accurately. The exclusion criteria were severe aortic stenosis, anticipated cardiac transplantation, and use of a ventricular assist device.

**FIGURE 1:**
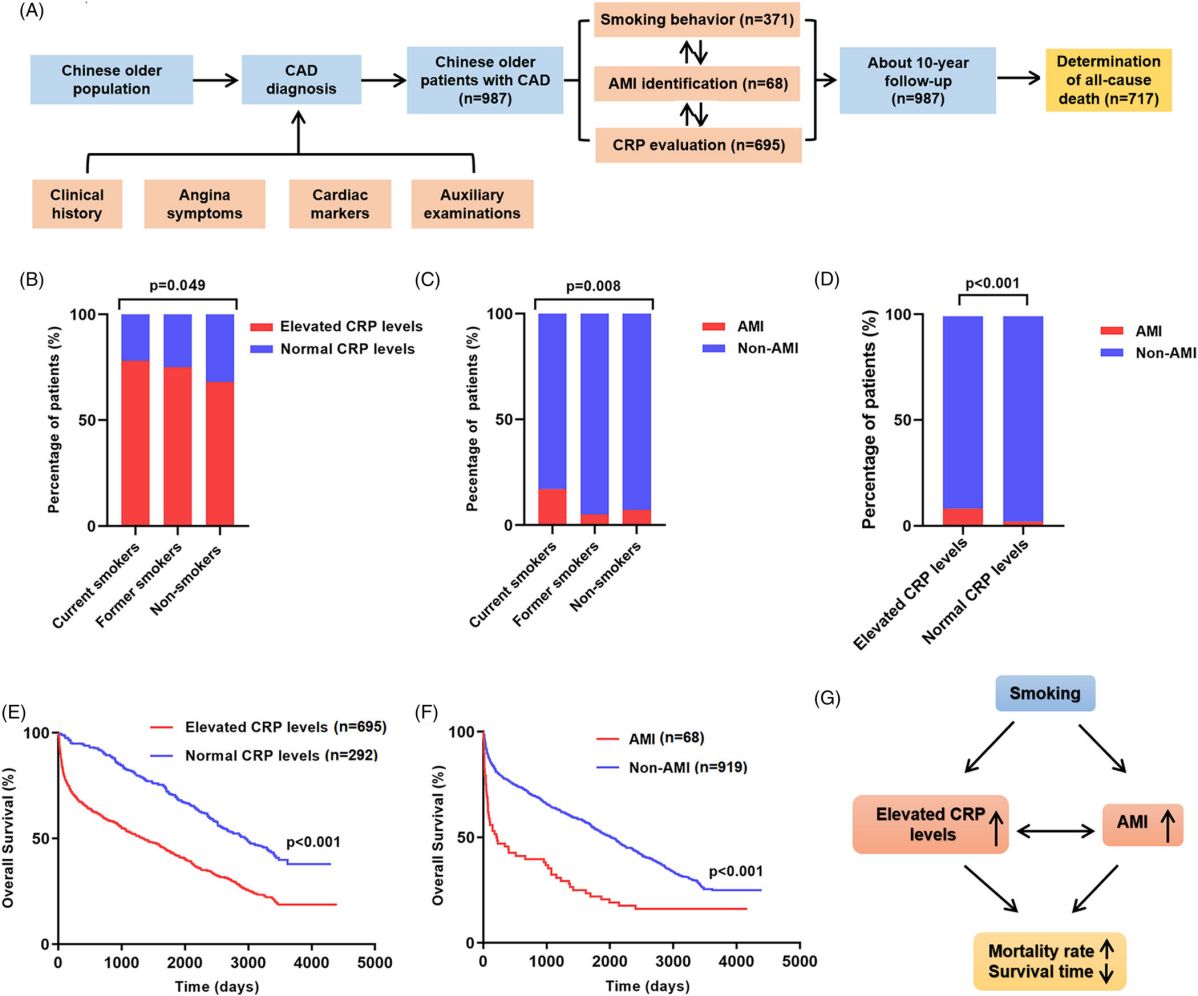
Relationships between smoking behavior, systemic inflammation, and myocardial infarction and their effects on long‐term outcomes in older Chinese patients with coronary artery disease (CAD). (A) Flow chart of this study. (B) Current smokers and former smokers had higher proportions of elevated C‐reactive protein (CRP) levels (75.9% and 74.8%, respectively) than non‐smokers (67.7%; *p* = 0.049). (C) Current smokers had a higher proportion of acute myocardial infarction (AMI) than former smokers and non‐smokers (16.7%, 5.0%, and 7.0%, respectively; *p* = 0.008). (D) 8.9% of patients with elevated CRP levels had AMI, whereas 2.1% of patients with elevated CRP levels had AMI (*p* < 0.001). (E) Kaplan–Meier analysis showing that patients with elevated CRP levels had lower overall survival (*p* < 0.001). (F) Kaplan–Meier analysis showing that patients with AMI had lower overall survival (*p* < 0.001). (G) Positive relationships between smoking behavior, elevated CRP levels, and AMI, and positive relationships between elevated CRP levels, AMI, and long‐term mortality.

On the basis of smoking behavior, patients were classified as current smokers, former smokers, or non‐smokers. Current smokers were defined as having smoked more than one cigarette per day for the last year. Former smokers were those who had a history of cigarette use on a regular basis (more than one cigarette per day) but were not current smokers. AMI was diagnosed by the chief physicians according to the 2007 version of the Universal Definition of Myocardial Infarction.^4^ Blood samples of all participants were collected to measure CRP levels at the central laboratory of the Department of Biochemistry, Chinese PLA General Hospital. According to Ridker,^5^ elevated CRP levels were defined as >0.2 mg/dL, above which a low level of systemic inflammation is generally considered.

Follow‐up lasted for approximately 10 years, and no patient was lost to follow‐up. Follow‐up data were obtained from medical records and telephone interviews. Death was determined from death records and legal documents, including time, site, and other information. We conducted a follow‐up to evaluate all‐cause mortality within an average period of 1836 days (median: 1871 days; interquartile range: 384−3225 days).

The median age of the 987 patients was 86 years (range: 60−104 years). Table S1 presents the characteristics of patients with CAD. Patients with smoking behavior, which included current smokers and former smokers, had higher proportions of elevated CRP levels (75.9% and 74.8%, respectively) than non‐smokers (67.7%; *p* = 0.049; Figure 1B). Smoking behavior had positive correlations with elevated CRP levels (*r* = 0.075; *p* = 0.018). Moreover, current smokers had a higher proportion of AMI than former smokers and non‐smokers (16.7%, 5.0%, and 7.0%, respectively; *p* = 0.008; Figure 1C). Current smokers had a positive correlation with AMI (*r* = 0.111; *p* = 0.006). Current smokers were more likely to have elevated CRP levels with an odds ratio (OR) of 1.970 (95% confidence interval [CI]: 1.029−4.008; *p* = 0.049; Table S2) in multivariable logistic regression analysis. Furthermore, current smokers were more likely to have AMI with an OR of 2.780 (95% CI: 1.154−6.188; *p* = 0.016) in multivariable logistic regression analysis.

A total of 8.9% of patients with elevated CRP levels had AMI, whereas 2.1% of patients without elevated CRP levels had AMI (*p* < 0.001; Figure 1D). Patients with elevated CRP levels were more likely to be current smokers, former smokers, and had AMI (*p* < 0.05; Table S1). Patients with AMI were more likely to be current smokers and had elevated CRP levels (*p* < 0.05). Elevated CRP levels had a positive correlation with AMI (*r* = 0.124; *p* = 0.001). Patients with elevated CRP levels were more likely to have AMI with an OR of 4.398 (95% CI: 2.013−11.580; *p* < 0.001; Table S2) in multivariable logistic regression analysis.

In total, 717 deaths (72.6%) occurred during the 10‐year follow‐up period. Dead patients had more elevated CRP levels and AMI (*p* < 0.05; Table S1). Elevated CRP levels and AMI were positively associated with mortality, with ORs of 1.928 (95% CI: 1.623−2.302) and 2.222 (95% CI: 1.664−2.910) in multivariable Cox regression analyses, respectively (*p* < 0.001; Table S3). Patients with elevated CRP levels or AMI had lower overall survival (*p* < 0.001; Figure 1E,F).

The relationships between smoking behavior, systemic inflammation, AMI, and mortality have been previously reported. However, they usually focus on the general population and do not have a detailed analysis of older people. Thus, we conducted a 10‐year follow‐up study on 987 older Chinese patients with CAD and evaluated the relationships between smoking behavior, elevated CRP levels, AMI, and mortality. This study found that smoking behavior was positively and significantly associated with elevated CRP levels and AMI and played an important role in affecting elevated CRP levels and AMI. We also confirmed that elevated CRP levels were positively associated with AMI as a potential biomarker for identifying AMI and that elevated CRP levels and AMI increased mortality and reduced survival in older Chinese patients with CAD (Figure 1G).

Smokers tend to have elevated CRP levels among subjects with an average age of 61.5 years.^2^, ^3^ This study illustrated that in older CAD patients with a median age of 86 years, current smokers and former smokers had elevated CRP levels compared with non‐smokers. Meanwhile, current smokers had a higher proportion of AMI than former smokers and non‐smokers. Moreover, patients with elevated CRP levels had a higher proportion of AMI. Data show that systemic inflammation has an adverse influence on atherosclerosis.^2^, ^3^ In this study, elevated CRP levels affected mortality, consistent with previous studies.^2^, ^3^ Our results suggest that CRP could also be used as a good biomarker for AMI identification and long‐term outcomes in older Chinese patients with CAD.

This prospective study demonstrated positive relationships between smoking behavior, elevated CRP levels, and AMI, and positive relationships between elevated CRP levels, AMI, and long‐term mortality in older Chinese patients with CAD. Smoking behavior could play an important role in affecting systemic inflammation represented by elevated CRP levels and subsequent development of AMI caused by systemic inflammation. As the recognized biomarker of systemic inflammation, CRP could also be used as a good biomarker for AMI identification and long‐term outcomes, whereas AMI increases long‐term mortality and reduces long‐term survival in older Chinese patients with CAD.

## AUTHOR CONTRIBUTIONS

N.L. contributed to the study design, conducted the data analyses, and drafted the paper. Y.S., H.Z., and Q.L. conducted the data collection and analyses. P.Y. and P.P. contributed to the study design and reviewed the paper. Y.Z. and S.F. contributed to the study design, collected the data, and reviewed the paper. All authors read and approved the final manuscript.

## CONFLICT OF INTEREST STATEMENT

The authors declare no conflicts of interest.

## FUNDING INFORMATION

This work was supported by grants from the Military Medical Science and Technology Youth Incubation Program (20QNPY110), National Natural Science Foundation of China (81900357), Excellent Youth Incubation Program of Chinese PLA General Hospital (2020‐YQPY‐007), Natural Science Foundation of Hainan Province (821QN389), National Key R&D Program of China (2018YFC2000400), National S&T Resource Sharing Service Platform Project of China (YCZYPT[2018]07), Specific Research Fund of Innovation Platform for Academicians of Hainan Province (YSPTZX202216), and Technology Program of Hainan Province (ZDKJ2019012). The sponsors had no role in the design, conduct, interpretation, review, approval, or control of this study.

## ETHICS STATEMENT

The study protocol was approved by the Ethics Committee of Chinese PLA General Hospital (Beijing, China; Number: 038) and was implemented in accordance with the Declaration of Helsinki (as revised in 1983). All participants provided informed consent.

## Supporting information

Supporting information

## Data Availability

All data and material are available upon reasonable request to the corresponding authors.
