# Supplementary material for: Relationships between smoking behavior, systemic inflammation, and myocardial infarction and their effects on long‐term outcomes in older Chinese patients with coronary artery disease: A prospective study with a 10‐year follow‐up
Source: MedComm (2020). 2023 Apr 27;4(3):e240. doi: 10.1002/mco2.240 (PMC10140367; doi:10.1002/mco2.240)
Supplement: Supplementary file 1 — Supporting information [file MCO2-4-e240-s001.docx]

**Relationships between smoking behavior, systemic inflammation, and myocardial infarction and their effects on long-term outcomes in older Chinese patients with coronary artery disease: A prospective study with a 10-year follow-up**

Nanhang Lei^1,＃^ Yijie Sun^1,＃^ Hanwang Zhou^1,＃^ Qiong Liu^1,＃^ Yali Zhao^1,*^ Pengbin Yin^2,*^ Ping Ping^3,*^ Shihui Fu^1,*^

^1^Hainan Hospital of Chinese People’s Liberation Army General Hospital, Sanya, China

^2^Chinese People’s Liberation Army General Hospital, Beijing, China

^3^General Station for Drug and Instrument Supervision and Control, Beijing, China

^*^**Correspondence**

Shihui Fu

Hainan Hospital of Chinese People’s Liberation Army General Hospital, Sanya, China. Email: [xiaoxiao0915@126.com](mailto:tjssqr.2000@163.com)

Ping Ping

General Station for Drug and Instrument Supervision and Control, Beijing, China. Email: [pingping301@126.com](mailto:sci6688@126.com;)

Pengbin Yin

Chinese People’s Liberation Army General Hospital, Beijing, China.

Email: [yinpengbin@gmail.com](mailto:luoleiming@vip.sina.com.)

Yali Zhao

Hainan Hospital of Chinese People’s Liberation Army General Hospital, Sanya, China. Email: [zhaoyl301@163.com](mailto:zhaoyi301@163.com)

^＃^Co-first authors.

Emails:

Nanhang Lei: nanhangray@foxmail.com

Yijie Sun: [824892785@qq.com](mailto:chenwenjidr@163.com)

Hanwang Zhou: zhouhanwang2021@163.com

Qiong Liu: liuqiong301hp@126.com

Yali Zhao: [zhaoyl301@163.com](mailto:zhaoyi301@163.com)

Pengbin Yin: [yinpengbin@gmail.com](mailto:luoleiming@vip.sina.com.)

Ping Ping: [pingping301@126.com](mailto:sci6688@126.com;)

Shihui Fu: xiaoxiao0915@126.com.

| **Table S1.** Characteristics of older patients with CAD | | | | | | | | | | |
| --- | --- | --- | --- | --- | --- | --- | --- | --- | --- | --- |
| **Characteristics** | **Total   (n = 987)** | **Elevated CRP**  **levels**  **(n = 695)** | **Normal CRP**  **levels**  **(n = 292)** | **P-value** | **With AMI (n = 68)** | **Without AMI (n = 919)** | **P-value** | **Death  (n = 717)** | **Survival  (n = 270)** | **P-value** |
| Age (years)^a^ | 86 (82–90) | 86 (82–90) | 86 (81–89) | 0.124 | 87 (84–90) | 86 (82–90) | 0.102 | 88 (84–91) | 82 (76–85) | <0.001 |
| Male (%) | 887 (89.9) | 618 (89.0) | 269 (91.8) | 0.206 | 64 (94.1) | 823 (89.6) | 0.229 | 647 (90.2) | 240 (88.9) | 0.531 |
| BMI (Kg/m^2^)^a^ | 24.2 (21.7–26.6) | 24.5 (21.7–27.0) | 24.8 (22.5–27.6) | 0.071 | 23.2 (21.0–26.0) | 24.3 (21.8–26.6) | 0.078 | 24.0 (21.5–26.4) | 24.7 (23.0–26.7) | 0.001 |
| Smoking behavior (%) |  |  |  | 0.049 |  |  | 0.008 |  |  | 0.081 |
| Current smokers (%) | 54 (5.5) | 41 (5.9) | 13 (4.5) |  | 9 (13.2) | 45 (4.9) |  | 33 (4.6) | 21 (7.8) |  |
| Former smokers (%) | 317 (32.1) | 237 (34.1) | 80 (27.4) |  | 16 (23.5) | 301 (32.8) |  | 244 (34.0) | 73 (27.0) |  |
| AMI (%) | 68 (6.9) | 62 (8.9) | 6 (2.1) | <0.001 |  |  |  | 57 (7.9) | 11 (4.1) | 0.034 |
| Hypertension (%) | 792 (80.2) | 556 (80.0) | 236 (80.8) | 0.767 | 58 (85.3) | 734 (79.9) | 0.344 | 576 (80.3) | 216 (80.0) | 0.906 |
| Diabetes mellitus (%) | 295 (29.9) | 235 (33.8) | 60 (20.5) | <0.001 | 27 (39.7) | 268 (29.2) | 0.067 | 247 (34.4) | 48 (17.7) | <0.001 |
| Atrial fibrillation (%) | 346 (35.1) | 269 (38.7) | 77 (26.4) | <0.001 | 34 (50.0) | 312 (33.9) | 0.012 | 301 (42.0) | 45 (16.7) | <0.001 |
| Chronic heart failure (%) | 389 (39.4) | 280 (40.3) | 109 (37.3) | 0.385 | 33 (48.5) | 356 (38.7) | 0.111 | 299 (41.7) | 90 (33.3) | 0.017 |
| Chronic kidney disease (%) | 206 (20.9) | 153 (22.0) | 53 (18.2) | 0.173 | 8 (11.8) | 198 (21.5) | 0.063 | 164 (22.9) | 42 (15.6) | 0.012 |
| Elevated CRP levels (%) | 695 (70.4) |  |  |  | 62 (91.2) | 633 (68.9) | <0.001 | 550 (76.7) | 145 (53.7) | <0.001 |
| Survival time (days)^a^ | 1871  (384–3225) | 1864  (403.5–3207.0) | 1912 (312–3247) | 0.796 | 213  (43.5–1568) | 2025  (493–3233) | <0.001 | 1021 (156.0–2153) | 3363  (3242–3542) | <0.001 |
| Notes: ^a^median (interquartile range).  Abbreviations: CAD: coronary artery disease; CRP: C-reactive protein; AMI: acute myocardial infarction; BMI body mass index. | | | | | | | | | | |

**Supplementary tables**

| **Table S2.** Relationships between smoking behavior, elevated CRP levels, and AMI in older patients with CAD | | | |
| --- | --- | --- | --- |
| **Characteristics** | **OR**^a^ | **95% CI** | **P-value** |
| Smoking behavior and elevated CRP levels |  |  |  |
| Current smokers | 1.970 | 1.029 to 4.008 | 0.049 |
| Former smokers | 1.514 | 1.097 to 2.071 | 0.012 |
| Smoking behavior and AMI |  |  |  |
| Current smokers | 2.780 | 1.154 to 6.188 | 0.016 |
| Former smokers | 0.664 | 0.351 to 1.204 | 0.190 |
| Elevated CRP levels and AMI |  |  |  |
| Elevated CRP levels | 4.398 | 2.013 to 11.580 | <0.001 |
| Notes: ^a^multivariable logistic regression analyses adjusted for age, gender, body mass index, hypertension, diabetes mellitus, atrial fibrillation, chronic heart failure, and chronic kidney disease. Abbreviations: CRP: C-reactive protein; AMI: acute myocardial infarction; CAD: coronary artery disease; OR: odds ratio; CI: confidential interval. | | | |

| **Table S3.** Relationships between elevated CRP levels, AMI, and mortality in older patients with CAD | | | | | | |
| --- | --- | --- | --- | --- | --- | --- |
| **Variables** | **OR**^a^ | **95% CI** | **P-value** | **OR**^a^ | **95% CI** | **P-value** |
| Age | 1.093 | 1.078 to 1.108 | <0.001 | 1.095 | 1.080 to 1.110 | <0.001 |
| Male | 1.131 | 0.883 to 1.472 | 0.345 | 1.088 | 0.8513 to 1.413 | 0.513 |
| BMI | 0.970 | 0.945 to 0.986 | 0.001 | 0.975 | 0.954 to 0.996 | 0.020 |
| AMI |  |  |  | 2.222 | 1.664 to 2.910 | <0.001 |
| Hypertension | 0.843 | 0.700 to 1.022 | 0.076 | 1.221 | 1.007 to 1.471 | 0.038 |
| Diabetes mellitus | 1.232 | 1.058 to 1.434 | 0.007 | 1.182 | 1.014 to 1.377 | 0.032 |
| Atrial fibrillation | 1.156 | 0.964 to 1.379 | 0.113 | 1.207 | 1.005 to 1.442 | 0.041 |
| Chronic heart failure | 1.876 | 1.587 to 2.212 | <0.001 | 1.880 | 1.592 to 2.214 | <0.001 |
| Chronic kidney disease | 1.574 | 1.347 to 1.836 | <0.001 | 0.640 | 0.548 to 0.748 | <0.001 |
| Elevated CRP levels | 1.928 | 1.623 to 2.302 | <0.001 |  |  |  |
| Notes: ^a^multivariable Cox regression analyses adjusted for age, gender, body mass index, hypertension, diabetes mellitus, atrial fibrillation, chronic heart failure, and chronic kidney disease. Abbreviations: CRP: C-reactive protein; AMI: acute myocardial infarction; CAD: coronary artery disease; OR: odds ratio; CI: confidential interval; BMI body mass index. | | | | | | |

**Materials and Methods**

**Study population**

This study consecutively enrolled 987 patients with CAD who are ≥60 years old and were admitted to the Department of Geriatric Cardiology, Chinese People’s Liberation Army (PLA) General Hospital. As the designated hospital for all these patients, Chinese PLA General Hospital provided long-term and comprehensive medical services and kept clinical information and death records, thus making it easier for us to follow these patients effectively and judge the endpoints accurately. According to the guidelines of the American College of Cardiology (ACC), American Heart Association (AHA), and European College of Cardiology (ESC), CAD was diagnosed by chief physicians on the basis of clinical histories, angina symptoms, cardiac markers, and auxiliary examinations, including electrocardiogram (rest and exercise), echocardiography, radionuclide imaging, computed tomography, and coronary angiography. All examinations were conducted by well-trained clinicians, and the final identification was made by the chief physicians. The exclusion criteria were severe aortic stenosis, anticipated cardiac transplantation, and use of a ventricular assist device.

**Study variables**

Body mass index was calculated as weight (kg) divided by the square of height (m). On the basis of smoking behavior, patients were classified as current smokers, former smokers, or non-smokers. Current smokers were defined as having smoked more than one cigarette per day for the last year. Former smokers were those who had a history of cigarette use on a regular basis (more than one cigarette per day) but were not current smokers. Blood sample of all participants was collected to measure CRP levels at the central laboratory of the Department of Biochemistry, Chinese PLA General Hospital. According to Ridker, elevated CRP levels were defined as >0.2 mg/dL, above which a low level of systemic inflammation is generally considered.

AMI was diagnosed by the chief physician according to the 2007 version of Universal Definition of Myocardial Infarction. Patients with a systolic blood pressure ≥ 140 mmHg, with a diastolic blood pressure ≥ 90 mmHg, or receiving medication for the treatment of hypertension were defined as having hypertension. Patients with a fasting glucose concentration ≥ 7 mmol/L or receiving treatment with an oral hypoglycemic agent/insulin were defined as having diabetes mellitus. Atrial fibrillation and chronic heart failure were diagnosed by the chief physicians according to the ACC, AHA, and ESC guidelines for atrial fibrillation and the ESC guidelines for chronic heart failure. Chronic kidney disease was defined as glomerular filtration rate < 60 mL/minute/1.73 m^2^ on the basis of the definition of the Kidney Disease Outcomes Quality Initiative Working Group.

**Outcome evaluation**

Professional researchers conducted logistical checks and data re-evaluations to confirm the accuracy of all information recorded by other researchers. Owing to increased multiple organ failure in older patients and the priority of all-cause mortality in outcome studies, the primary endpoint evaluated in this study was all-cause mortality. Follow-up lasted for approximately 10 years, and no patient was lost to follow-up. Follow-up data were obtained from medical records and telephone interviews. Death was determined from death records and legal documents, including time, site, and other information. We conducted a follow-up to evaluate all-cause mortality within an average period of 1836 days (median: 1871 days; interquartile range: 384–3225 days).

**Statistical methods**

All analyses were performed using GraphPad Prism 9.3 software (GraphPad Software, Inc., La Jolla, CA, USA). The mean and standard deviation (normal distributions) or median and interquartile range (skewed distributions) were calculated for continuous variables. Categorical variables were documented as percentages of participants. The student’s t–test or non–parametric test (Mann–Whitney U test or Kruskal–Wallis test) was used to compare the differences between continuous variables with normal distributions or skewed distributions. The chi-square test or Fisher’s exact test (if the chi-square test was not satisfied) was used to compare the differences between categorical variables. Multivariable logistic regression analysis was used to observe the relationships between smoking behavior, elevated CRP levels, and AMI. Multivariable Cox regression analysis was used to estimate the relationships between elevated CRP levels, AMI, and mortality. Kaplan–Meier analysis was used to determine the effect on overall survival. Statistical significance was set at *P* < 0.05.
